# Editorial: Community series in post-translational modifications of proteins in cancer immunity and immunotherapy, volume II

**DOI:** 10.3389/fimmu.2023.1289016

**Published:** 2023-09-26

**Authors:** Naoe Taira Nihira, Tomohiko Ohta, Zichuan Liu, Xiangpeng Dai

**Affiliations:** ^1^ Department of Translational Oncology, St. Marianna University Graduate School of Medicine, Kawasaki, Japan; ^2^ School of Pharmaceutical Science and Technology, Tianjin University, Tianjin, China; ^3^ Key Laboratory of Organ Regeneration and Transplantation of Ministry of Education, First Hospital of Jilin University, Changchun, China; ^4^ National-Local Joint Engineering Laboratory of Animal Models for Human Disease, First Hospital of Jilin University, Changchun, China

**Keywords:** post-translational modifications, PD-1, PD-L1, tumorigenesis, cancer metabolisms

The emergence of immune checkpoint inhibitors (ICIs) allows cancer immunotherapy to move to the frontiers of cancer therapy. ICIs targeting CTLA-4, PD-1, and PD-L1 are applied to patients with advanced malignancies and prolong their survival. Among the ICIs, therapeutic antibodies against the PD-1/PD-L1 improve the clinical outcome, supporting the evidence that PD-1/PD-L1 axis strongly contributes to anti-tumor immunity. Nevertheless, it is also reported that some cancer patients exhibit limited response rates and resistance to ICIs, therefore restricting the clinical application of ICIs. To overcome this issue, elucidating molecular mechanisms underlying cancer immunity and optimizing the combination therapy of ICIs with other therapeutic strategies using molecular target drugs would be required. Especially, the enzymes that catalyze post-translational modifications (PTMs) would be promising molecular targets for immunotherapy or combined therapy. During the past few years, immune checkpoint molecules, including TIM-3, LAG-3, and TIGIT, have been newly identified. However, most papers listed in this Research Topic focused on PTMs of PD-L1. Therefore, we summarized the current pieces of evidences on PTMs of PD-L1 in this editorial.

## PTMs in regulating the PD-L1 expression

The PD-L1 stability was regulated by many E3 ligases and deubiquitinases, since PD-L1 expression is tightly regulated in a context-dependent manner. To date, the E3 ubiquitin ligases β-TRCP, SPOP, STUB1, HRD1, DCUN1D, NEDD4, RNF144A, c-Cbl, and ARIH1 were reported to regulate PD-L1 expression. Contrary, it is reported that PD-L1 can be deubiquitinated by CSN5, USP22, USP7, USP9X, and OTUB1 (Feng et al.).

### Cell cycle

PD-L1 expression fluctuates during the cell cycle; it increases in the M and early G1 phases and rapidly decreases in the late G1 and S phases. SPOP promotes the rapid reduction by the poly-ubiquitination of PD-L1. Since Cyclin D/CDK4 enhances the PD-L1 ubiquitination, CDK4/6 inhibitors, Palbociclib and Ribociclib, increase PD-L1 expression and enhance the efficacy of anti-PD-1 immunotherapy *in vivo* (1).

### Inflammatory stimulation

Upon TNFα stimulation, NF-κB up-regulates expression of deubiquitinase CSN5, resulting in increased PD-L1 expression without affecting the transcription level of PD-L1 (2). Curcumin, a CSN5 inhibitor, can be used with anti-CTLA4 antibody together to decrease PD-L1 level and inhibit PD-L1 to enhance the efficacy of immunotherapy (2). Therefore, manipulation of PD-L1 stability could be promising strategy for increased efficacy of combination treatment with immunotherapy.

### Cancer metabolisms

**Figure 1:**
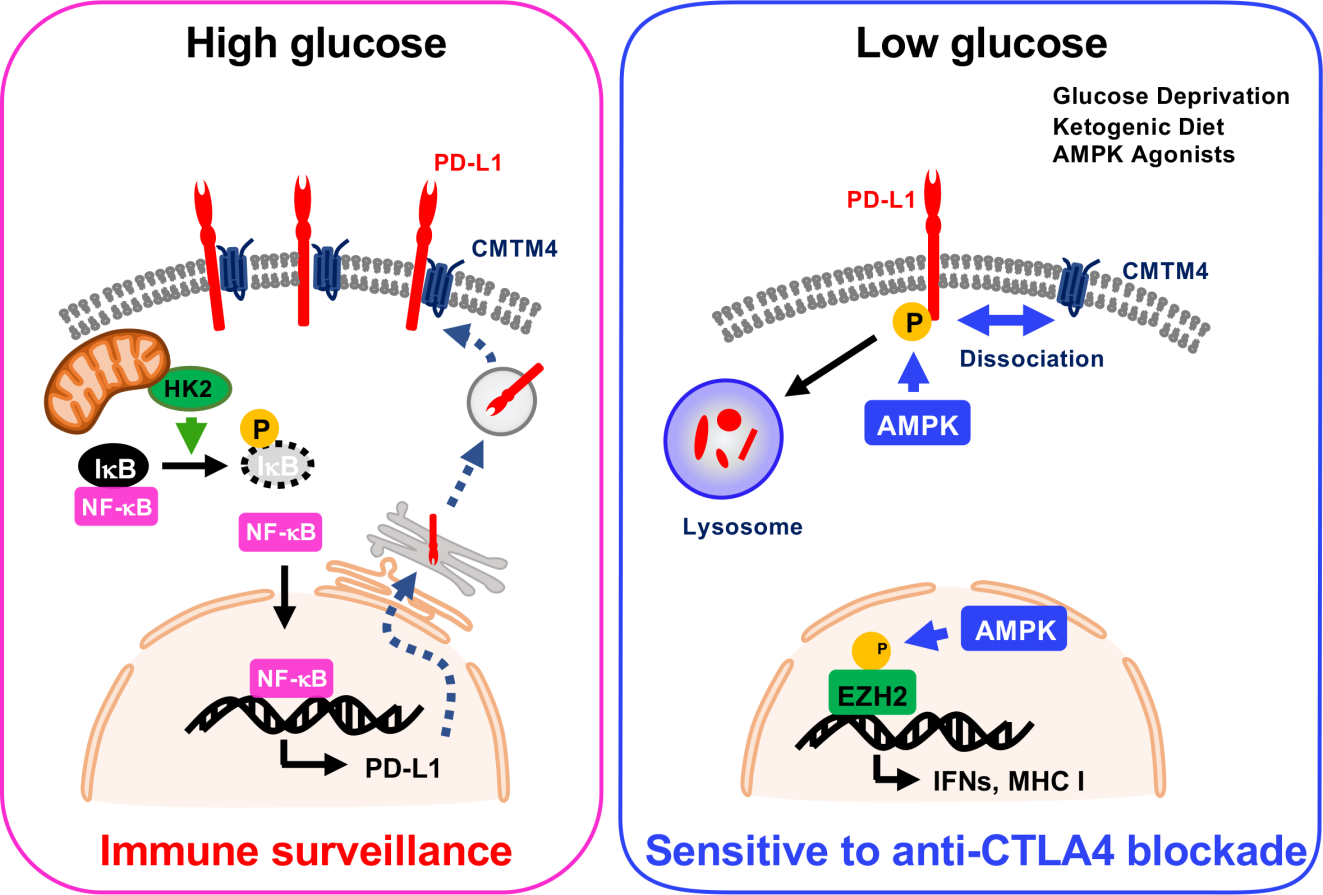
PTMs in the regulation of PD-L1 expression in cancer metabolism.

Dysregulation of cellular metabolism is one of the hallmarks of cancer progression, which was defined by Hanahan and Weinberg in 2011. Two independent studies demonstrated that energy metabolism regulates PD-L1 status at both transcriptional and post-translational levels. Dai et al. demonstrated that energy deprivation activates AMPK kinase, which subsequently phosphorylates PD-L1 at Ser283, resulting in PD-L1 degradation (3). CMTM4 and CMTM6 are critical regulators for lysosomal degradation of PD-L1. Mechanistically, phospho-mimic PD-L1 S283D mutation or treatment with AMPK-specific agonist A-769662 markedly attenuated the interaction between PD-L1 and CMTM4, and the dissociation triggers lysosomal degradation. Simultaneously, AMPK induces the expression of IFN- and antigen presentation-related genes through phosphorylation of EZH2 (3) (**Figure 1**). Furthermore, the mouse tumor model showed that ketogenic diet- or A-769662-induced PD-L1 degradation enhanced the CTLA-4 immune checkpoint blockade efficacy (3).

On the other hand, Lin et al. showed that high glucose condition increases expression of PD-L1 depending on NF-κB activation. Glycolytic enzyme hexokinase 2 (HK2) activation induced by high concentration of glucose phosphorylates IκBα at Thr291 and triggers its degradation, and then NF-κB translocates into the nucleus to upregulate PD-L1 transcription (**Figure 1**). In cancer cells, glucose uptake and the production of lactate are dramatically increased due to the expression of glucose transporters (GLUTs) and activation of glucose-responsible kinases. This characteristic might increase *PD-L1* expression and trigger immune surveillance in tumors.

## PTMs regulating the cellular distribution of PD-L1

Not only the expression control, the subcellular localization of PD-L1 is also tightly regulated by PTMs. After translation in the endoplasmic reticulum, translocation of newly synthesized PD-L1 to the plasma membrane from Golgi is regulated by K63-linked ubiquitination by mind bomb homolog 2 (MIB2) (4). Yu et al. screened the E3 ubiquitin ligases using shRNA library and found that knockingdown of *MIB2* reduces PD-L1 expression on the plasma membrane without affecting the total amount of PD-L1 (4). K63-linked poly-ubiquitination of PD-L1 at Lys136 in trans-Golgi network triggers RAB-8-mediated exocytosis, and then expresses on the plasma membrane (4).

On the other hand, several research groups reported that small portions of PD-L1 function in the cytoplasm and nucleus. Notably, Lys263 residue within PD-L1 is constitutively acetylated by acetyltransferase p300 (5). HDAC2-mediated PD-L1 deacetylation enables it to bind with regulators of sub-cellular localization, including Adaptin β2, Clathrin heavy chain, Vimentin, and Importin α1. RNA sequence analysis reveals that the expression of immune response-, NF-κB signaling-, type I IFN-, and type II IFN-related genes are induced by nuclear PD-L1 (5). It was reported that inhibition of nuclear translocation of PD-L1 by HDAC2 inhibitor increases the efficacy of anti-PD-1immunotherapy in mouse tumor models (5). To date, numerous HDAC inhibitors have developed and exhibited improved outcomes for cancer patients (Lian et al.). Among HDAC inhibitors, a class I HDAC inhibitor, Romidepsin, in combination with PD-1 blockade increases the efficacy of immunotherapy in lung adenocarcinoma. In addition, Entinostat is also implied in the combination treatment with ICIs (Lian et al.).

## Novel PTM regulating the tumor immunity

Aerobic glycolysis is also an important feature of cancer metabolism, which is well-known as the Warburg effect. Production of aerobic glycolysis, lactate, promotes the proliferation of tumor-related immune cells to build an immunosuppressive tumor microenvironment (TME). It is newly reported that protein lactylation is involved in the regulation of gene expression, which is considered the other function of lactate. Histone lactylation on Lys residues functions as an epigenetic hallmark during macrophage polarization (6). Thus, protein lactylation might be an essential biological event that links cancer metabolism to tumor immunity. Yang et al. compared the differentially expressed genes in tumor versus normal tissue and identified 11 lactylation-related genes. Among the 11 candidate genes, *HIBCH* expression is negatively correlated with tumor grade and TME score. Furthermore, the expression of *CTLA-4* and *PD-1* is also negatively linked with *HIBCH* expression, suggesting that HIBCH would be a potential biomarker to speculate ICI response.

## Conclusion

This Research Topic provides an overview of PTMs of PD-L1 and the combination therapy with ICIs. Further research about PTMs of PD-L1 must expand the choice of drugs that can be combined with ICIs.

## Author contributions

NN: Writing – original draft, Writing – review & editing. TO: Writing – original draft, Writing – review & editing. ZL: Writing – original draft, Writing – review & editing. XD: Writing – original draft, Writing – review & editing.
